# Novel lineages of single-stranded DNA phages that coevolved with the symbiotic bacteria *Rhizobium*

**DOI:** 10.3389/fmicb.2022.990394

**Published:** 2022-09-13

**Authors:** Jannick Van Cauwenberghe, Rosa I. Santamaría, Patricia Bustos, Víctor González

**Affiliations:** ^1^Centro de Ciencias Genómicas, Universidad Nacional Autonóma de México, Cuernavaca, Mexico; ^2^Department of Integrative Biology, University of California, Berkeley, CA, United States

**Keywords:** *Rhizobium*, phages, *Microviridae*, MCP, viral clusters, coevolution

## Abstract

This study describes novel single-stranded DNA phages isolated from common bean agriculture soils by infection of the nitrogen-fixing symbiotic bacteria *Rhizobium etli* and *R. phaseoli*. A total of 29 phages analyzed have 4.3–6 kb genomes in size and GC 59–60%. They belong to different clades unrelated to other *Microviridae* subfamilies. Three-dimensional models of the major capsid protein (MCP) showed a conserved β-barrel structural “jelly-roll” fold. A variable-length loop in the MCPs distinguished three *Rhizobium* microvirus groups. *Microviridae* subfamilies were consistent with viral clusters determined by the protein-sharing network. All viral clusters, except for *Bullavirinae*, included mostly microviruses identified in metagenomes from distinct ecosystems. Two *Rhizobium* microvirus clusters, chaparroviruses, and chicoviruses, were included within large viral unknown clusters with microvirus genomes identified in diverse metagenomes. A third *Rhizobium* microvirus cluster belonged to the subfamily *Amoyvirinae*. Phylogenetic analysis of the MCP confirms the divergence of the *Rhizobium* microviruses into separate clades. The phylogeny of the bacterial hosts matches the microvirus MCP phylogeny, suggesting a coevolutionary history between the phages and their bacterial host. This study provided essential biological information on cultivated microvirus for understanding the evolution and ecological diversification of the *Microviridae* family in diverse microbial ecosystems.

## Introduction

Viruses are the most abundant, ubiquitous, and diverse biological entities on earth (Breitbart and Rohwer, 2005; Suttle, 2007; Dion et al., 2020). These obligatory pathogens infect all known taxa of organisms, but most viruses are specialized to infect bacteria. These viruses are called bacteriophages or phages and play a key role in the evolution and ecology of bacteria. Phages shape bacterial community structure through the lysis of diverse bacterial genera, species, or even strains, on which they are specialized because of coevolution (Poullain et al., 2008; Parsons et al., 2012; Weitz et al., 2013; Morella et al., 2018; Wang et al., 2019). Phages can also transduce bacterial genes by erroneously packing bacterial DNA in the viral capsid or when prophages are excised inaccurately from the host genome (Pedulla et al., 2003; Canchaya et al., 2004; Touchon et al., 2017). Prophages are phages integrated into the host genome, which can excise and induce lysis after multiple host generations or remain stranded as cryptic prophages when excision genes experience loss-of-function mutations (Casjens, 2003; Wang et al., 2010; Ramisetty and Sudhakari, 2019).

Single-stranded (ss) DNA viruses are less thoroughly studied and are a minor fraction of the phages found in databases, which abound in double-stranded (ds) DNA viruses (16.2% as of 2022; Roux et al., 2016a, 2016b). However, they appear to be prevalent in aquatic ecosystems and the human gut microbiome (López-Bueno et al., 2009; Shkoporov et al., 2019). The most common ssDNA viruses are *Microviridae*, composed of a small (~25 nm) capsid with icosahedral symmetry and a 4,000–6,500 bp genome (Maclean and Hall, 1962; Roux et al., 2012; Doore and Fane, 2016). *Bullavirinae* and *Gokushovirinae* are the two *Microviridae* subfamilies recognized by the International Committee on Taxonomy of Viruses (ICTV) based on structural and genomic differences (King et al., 2011). Other subfamilies suggested are *Aravirinae* (Quaiser et al., 2015), *Pichovirinae* (Roux et al., 2012), and *Alpavirinae* (Krupovic and Forterre, 2011). Most known *Bullavirinae* have been isolated infecting Enterobacteria and have been the subject of early research on phage biology. *Gokushovirinae* and suggested subfamilies are known to infect diverse bacterial taxa (Roux et al., 2012; Labonté and Suttle, 2013; Quaiser et al., 2015; Zheng et al., 2018).

*Microviridae* were long believed to be exclusively lytic, with the only exemption being the temperate *Alpavirinae* (Krupovic and Forterre, 2011). Until recently, some studies discovered prophages belonging to *Gokushovirinae* (Kirchberger and Ochman, 2020) and *Bullavirinae* (Kirchberger et al., 2021) through the analysis of host genomes. These findings demonstrate the limited understanding of the diversity and prevalence of *Microviridae* prophages. *Microviridae* have been detected in human and animal microbiomes and aquatic systems (Roux et al., 2012; Kirchberger and Ochman, 2020). In contrast, the diversity of phages infecting soil and rhizosphere-dwelling bacteria is poorly described (Zablocki et al., 2016; Pratama and van Elsas, 2018). Rhizobia is a particular group of bacteria adapted to the rhizosphere (Nautiyal, 1997; Wheatley et al., 2020). They engage in a mutualistic association with legumes, forming nodules on the roots and fixing atmospheric nitrogen in exchange for photosynthates. Rhizobia are infected by various families of *Caudovirales* (Werquin et al., 1988; Santamaría et al., 2022). In our recent study, we report that the *Microviridae* family likely includes a significant fraction of phage communities infecting rhizobia associated with common beans (Van Cauwenberghe et al., 2021).

Here, we aim to investigate the infection properties of *Rhizobium* microviruses and the structural features of the capsid surface. Moreover, we want to determine the phylogenetic relationship among *Microviridae* associated with rhizobia and the coevolutionary history of these phages with their hosts.

## Materials and methods

### Microvirus isolation and genome sequencing

Three phages, RHEph17, X92, and X94, originating from common bean agriculture soils in Mexico, were isolated by infection enrichment according to Santamaría et al. (2014) using *R. etli* N741, *R. etli* GR14, and *R. phaseoli* GR75 as host, respectively. Pure phages were obtained after plaquing three times in double-layer plates. Next, the phage genomic DNA was extracted following the next protocol: phages were propagated in 2 × 6 ml of PY broth, adding 0.1 ml of phage stock solution (PFUs 10^9^) to the host strain at OD_600_ at 0.1, and incubated overnight.

Next, the lysate was added to 10% v/v chloroform to remove cell debris and centrifuged to recover 10 ml of the supernatant. Next, the lysate was treated with 0.1 ml of DNAse (10 mg/ml; Roche Diagnostics) and RNase (10 mg/ml; MP Biomedicals) to remove the DNA and RNA of bacteria. Phages were precipitated with 12% w/v PEG8000 and 1 M NaCl overnight at 4°C, then centrifuged (30 min at 10,000 rpm). Finally, concentrated phage was treated with the DNA Isolation Kit for Cells and Tissues (Roche Life Sciences, CA, United States) following the procedure for DNA purification for gram-negative bacteria, adjusting the volumes used to 0.5 ml of a lysis solution with 10 μl of proteinase K, 20 μl RNase and 0.24 ml precipitation solution. DNA was precipitated with 0.7 volumes of isopropanol and 1 ml of 70% ethanol and resuspended in 0.1 ml water. Phage genome mobility and integrity were evaluated by agarose gel electrophoresis.

Genome sequencing was performed with pair-end libraries made with the Nextera Kit and sequenced in an Illumina NextSeq 500 sequencer at Unidad Universitaria de Secuenciación Masiva de DNA [UUSMD]-Universidad Nacional Autónoma de México [UNAM]. Readings of 135 bp were trimmed with TrimGalore^1^ and assembled using Spades v. 3.13.1, Velvet v. 1.2.10, and Phred/Phrap/Consed v. 23.0 (Zerbino and Birney, 2008; Bankevich et al., 2012). The assembled sequence demonstrated that the samples are small circular genomes of about 4.3–6.3 kb, according to their mobility in gel electrophoresis.

### Microvirus dataset

In addition to the three microviruses isolated in this work, we included 26 previously reported microviruses (Van Cauwenberghe et al., 2021; Supplementary Table S1). The current study describes the features of the 29 microviruses in this collection. These belong to three genomic clusters, defined by the average nucleotide identity based on MUMmer (ANIm; Pritchard et al., 2016) above 80%, provisionally referred to as F02, F08, and F40 (Van Cauwenberghe et al., 2021). The second set of microvirus genomes was obtained by downloading 2,147 genomes of *Microviridae* from GenBank. They consist of 1,605 microviruses identified in metagenomes, and 542 represent the genome sequence of isolated virions (Supplementary Table S2). Moreover, 25 recently published microvirus genomes were identified in metagenomic fecal samples of flying foxes (Lopez et al., 2022). Finally, they were incorporated into a local database to run the vConTACT v2 viral clustering method (Supplementary Table S3).

### Phage growth conditions and infection kinetics

*Rhizobium* strains were grown in peptone-yeast extract broth (PY added with 7 mM CaCl_2_ and 20 μg/ml nalidixic acid) in an incubator at 30°C with shaking at 200 rpm. Phage titer was estimated by mixing 200 μl of the corresponding host strain in the exponential growth phase and 100 μl of 10-fold phage dilutions with top agar (soft medium with 0.4% agar melted at 42°C) and poured onto plates with 1.5% agar. Double-layer plates were allowed to cool and were subsequently incubated at 30°C overnight.

Host-range determination was carried out with the spot-test technique range in double-layer plates (Hyman and Abedon, 2009). First, bacterial lawns were made by mixing 300 μl of strain with a soft medium and pouring it over PY-agar plates. Next, 10 μl of the phage solution from stocks were dropped onto the lawns and incubated at 30°C. After incubation, spots were registered as complete lysis (transparent plaque), partial lysis (translucent plaque), or resistance to lysis when no plaque was observed. R (v. 3.6.1) programming was used to perform box plots and two-sample T-test to assess the statistical significance of the host-range infection rates.

Infection kinetics analyses were performed in 96-well microplates in a BioTek Synergy 2 Multi-Mode Microplate Reader (Agilent, United States) using the *R. phaseoli* N2.5 strain as the host. Infections were tested at Multiplicities of Infection (MOI) of 1–0.00006. The change in optical density was tracked in time by measuring optical density every 30 min.

The one-step growth curve of phage TM23, TM24, and Y67 was determined using the *R. etli* N2.5 strain. Bacterial culture and phage were mixed in 1 ml medium at MOI of 0.01 (1 × 10^8^ bacterial cells and 1 × 10^6^ phages) and incubated for 30 min at 30°C to allow phage adsorption. Subsequently, the unabsorbed phage was removed by centrifugation, discarding the supernatant. Pellets with infected cells were diluted in 1 ml of fresh medium, transferred to a 250 ml flask with 100 ml of fresh PY broth, and incubated at 30°C. Samples were taken every 30 or 60 min for 8.5 h, and 10-fold dilutions were performed for plaquing. The experiment was performed in triplicate, and PFUs (plaque-forming units) were registered and plotted using ggplot2 (v. 3.3.6) in R (v. 3.6.1). Burst size was calculated by the difference between the average PFUs at the maximum virus release stage and the average PFUs at the eclipse period and by dividing that number by the number of infected cells (total phages minus unabsorbed phages).

### Electron microscopy

Phage samples were propagated in a 250 ml flask by adding 1 ml of phage solution from the stock (10^9^ PFUs/ml) in 100 ml of PY medium containing the corresponding host strain at an OD of 0.1 and incubated overnight. The lysate was then centrifuged (10 min at 10,000 rpm), passed through a 0.22-μm membrane filter, and subject to precipitation using 12% w/v PEG8000 and 1 M NaCl overnight at 4°C. Then, the solution was centrifuged (30 min at 10,000 rpm), and the pellet was resuspended in 5 ml 10 mM MgSO_4_. One volume of chloroform was added to remove PEG, and the mixture re-centrifuged. Subsequently, the aqueous phase was recovered, and phages were concentrated using an Amicon Ultra-15 100 K filter (Merck Millipore) to a volume of 0.2 ml. The phage titer was about 1 × 10^10^ PFUs/ml at the end of the purification. A droplet of these viral particles was negatively stained with 1% uranyl acetate on a copper grid covered with evaporated carbon and Formvar film (E.M.S. FF200-Cu). Electron microscopy was performed in a transmission electron microscope Libra 120 (Zeiss, Germany) coupled with a Multiscan Camera (GATAN Inc., United States) at the Unidad de Microscopía Electrónica, UNAM.

### Three-dimensional models of the major capsid protein (MCP)

The amino acid sequences of the MCPs of TM23, TM24, Y67, phiX174 (*Bullavirinae*), and SpV4 (*Gokushovirinae*) were used to obtain the three-dimensional models (3D) with AlphaFold-2.1.0 in ColabFold server using the MMseq2 algorithm (Jumper et al., 2021; Mirdita et al., 2021). The unrelaxed rank 1 models were visually inspected with the RSCB PDB 3D Viewer Mol* (Sehnal et al., 2021) and then were aligned in pairs to superimpose the 3D models using the matchmaker tool in UCSF Chimera X v. 1.2 software (Pettersen et al., 2021). The quality of the models was assessed first by the local Distance Difference Test (lDDT) method (Mariani et al., 2013) and second by the superposition of pairs of predicted structures, including the predicted 3D MCP models with the experimental structure of MCP- phiX174 (RCSB PDB Protein Data Bank, ID 2BPA; PDB DOI: 10.2210/pdb2BPA/pdb). High lDDT scores (>80) were assigned for the MCPs TM23, TM24, and Y67 at the equivalent structural regions in the phiX174 structure. The root mean square deviation (RMSD), which represents the dissimilarity of the predicted protein structures, was calculated by measuring the average distance between atoms of the proteins when superimposed. Lower RMSD values indicate a more significant similarity of predicted protein structures. The similarity between MCP 3D models was determined by calculating the distance matrix with the R package “dist” using the method “maximum.” The dendrogram was built with the R package “hclust” using the method “complete.” An additional comparison included the 3D model of the MCP of phage HK97 (*Caudovirales*) as a control.

### Comparative genomics

Average nucleotide identity (ANI) among phages described here and representative phages of different *Microviridae* subfamilies was calculated with pyani v.0.2.9 using the ANIm MUMmer method (Pritchard et al., 2016). In addition, average amino acid identity (AAI) was calculated with the script aai.rb from the enveomics collection (Rodriguez-R and Konstantinidis, 2016) and plotted with ggplot2 function heatmap 2. Gene-sharing networks were created using vConTACT v2 (Jang et al., 2019). First, the vConTACT v2 tool clusters similar proteins into protein clusters (PCs) using the Markov cluster algorithm (MCL). Then, viral clusters (VCs) were calculated according to the maximum probabilities of sharing PCs (edges) between the genomes (nodes) to produce a bipartite network. The VCs were defined using ClusterONE, with default parameters (MCL inflation: 2; penalty value: 2; edge weight: 10). The networks generated by vConTACT v2 were visualized using Cytoscape v3.8.2.^2^

### Phylogenetic analyses

Phylogenetic trees of *Microviridae* were constructed using the major capsid protein (MCP) identified in *Microviridae* isolated here and previously(Van Cauwenberghe et al., 2021), in previously suggested *Microviridae* subfamilies, and detected using BLASTp (e-value <10^−6^, % identity >70%, query cover >30%). An alignment of MCP was created using MUSCLE (Edgar, 2004). A maximum-likelihood phylogenetic tree was constructed with IQTREE with 1,000 bootstrap replicates, using the GTR+F+R6 model selected using the Bayesian Information Criterion (BIC; Nguyen et al., 2015).

Genome phylogeny of *R. etli* and *R. phaseoli* strains was based on concatenated 3,602 core proteins obtained with the Bacterial Pan Genome Analysis Tool (BPGA; Chaudhari et al., 2016). A protein super alignment was performed using MUSCLE (Edgar, 2004), and gaps were processed with TrimAI (Capella-Gutiérrez et al., 2009). The phylogenetic tree was constructed with IQTREE with 1,000 bootstrap replicates with the JTT+F+R3 model.

### Co-phylogeny of Microviridae prophages and their hosts

We used ParaFit (Legendre et al., 2002) in the ape package in R (Paradis and Schliep, 2019) to test for significant similarities between bacterial phylogenies (based on 16S nucleotide sequences) and the MCP phylogenies of both the 29 lytic *Microviridae* used in this study and MCPs located in bacterial genomes identified by BlastP at the species level or available on the NCBI RefSeq database.

Each tree was built using IQTREE using the LG+I+G4+F substitution model for phages, and the TIM3+I+G4+F substitution model for bacteria, with 1,000 bootstrap replicates. Models were selected using the Bayesian Information Criterion (BIC) implemented on the IQTREE web server (Nguyen et al., 2015). Patristic distances of the phylogenies were calculated using cophenetic.phylo from the ape package in R.

## Results

### Infection properties and host range of selected *Rhizobium* microviruses

**Figure 1:**
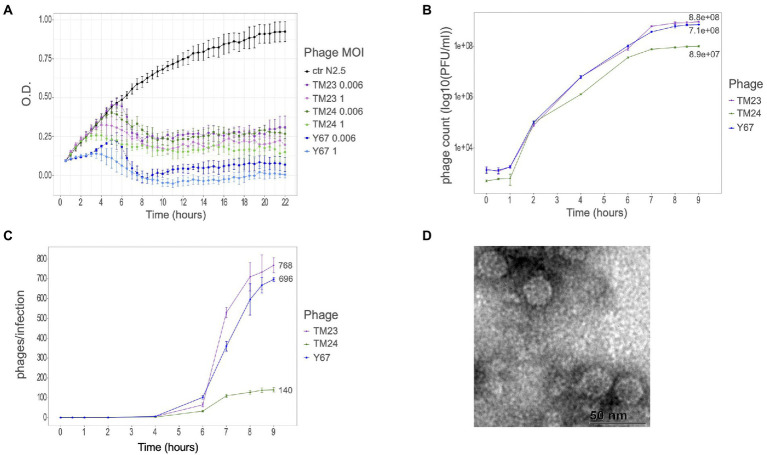
Infectious properties and morphology of *Rhizobium* microvirus. **(A)** Infection kinetics in PY-broth culture of phages TM23 (representing ANIm cluster F02), TM24 (F08), and Y67 (F40) at two different MOIs (1 and 0.006) on *R. etli* N2.5. **(B)** Phage titers after 9 h of infection on N2.5. **(C)** Two-step growth curve of the three phages at MOI 0.01. Colors code indicating the respective strains is at the right of the panels. **(D)** Electron micrograph of TM23 at 50 nm.

To investigate the infection features and host range of *Rhizobium* microviruses, we chose one representative phage per each of the already-described ANIm *Microviridae* clusters: F02, F08, and F40 (Van Cauwenberghe et al., 2021). Infection kinetics at different MOIs was performed using the *R. etli* strain N2.5 host (Figure 1A; Supplementary Figure S1). Although the three phages differ in the genome sequence, they are equally competent to lyse the N2.5 host. The phage Y67 (belonging to ANIm cluster F40) was very efficient to lyse cultures of N2.5, even at lower MOIs in comparison with TM24 (F08) and TM23 (F02). Despite slight differences in the infection kinetics of TM23 and TM24, they both eliminate the bacterial cells at the lowest MOI (0.00006) after 6 h of incubation. Moreover, the titer of phages after 9 h of incubation with N2.5 strain was moderately high for Y67 and TM23 (9 × 10^8^ and 7 × 10^8^ PFUs/ml, respectively) and low for TM24 (9 × 10^7^ PFUs/ml; Figure 1B).

To assess the infection features of *Rhizobium* microvirus, we did the one-step-growth kinetics for the three phages TM23, TM24, and Y67 (Figure 1B). The latent phase took about 4 h to yield the first virions in all the three phages; after 9 h, the virion production stopped in TM23 and Y67, but phage TM24 developed slow kinetics with a very short exponential phase (Figure 1C). The estimated burst size of TM23 and Y67 was 768 and 669 virions per cell, respectively. In contrast, TM24 had the smallest burst size of 140 virions per cell.

**Figure 2:**
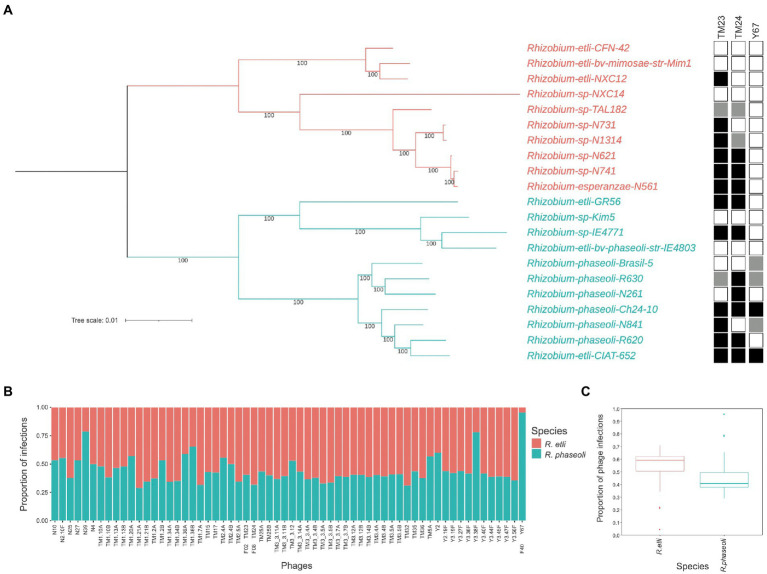
Host infection range of *Rhizobium* microvirus. **(A)** Maximum likelihood unrooted tree with the common core genome of *R. etli* and *R. phaseoli* strains and the infection by TM23, TM24, and Y67 in spot-assays. Black squares indicate complete lysis; gray, partial lysis; and empty squares, absence of lysis. **(B)** The proportion of infection of 63 microviruses on *R. etli* and *R. phaseoli* strains. **(C)** Box plots of the proportion of phage infections on the two *Rhizobium* species with the median and the quartile distribution. Data on infection for B and C were extracted from the phage-bacteria infection matrix reported in (Van Cauwenberghe et al., 2021, “Dataset 1”).

To look for species-specific infection preferences of these three phages, we compare their range of infection by spot-assays over a collection of 10 *R. etli* strains and 11 *R. phaseoli* strains whose complete genome is known and devoid of *Microviridae*-related sequences. Spot phenotypes appear as evident lysis (transparent), medium lysis (translucid), and resistant (no phenotype). TM23 and TM24 infection yielded transparent spots in 12 and 10 out of 21 strains (proportion 0.6 of *R. etli* over *R. phaseoli*) of both species without any observable species-specificity (Figure 2A). These spot phenotypes did not associate with the phylogenetic separation of the strains into the species *R. etli* and *R. phaseoli*. In contrast, the Y67 phage showed marked preference and limited host range for *R. phaseoli* strains (Figure 2A). The proportion of infection (clear spots) in 63 microviruses of the collection previously reported (Van Cauwenberghe et al., 2021) showed a significant trend to infect *R. etli* over *R. phaseoli* (two-samples t-test *p* = 2 × 10^−6^; Figures 2B,C). Moreover, the three *Rhizobium* microvirus tested produced partially lytic spots (translucid) in some strains, indicating differences in the efficiency of infection, an issue that deserves further experimentation.

### Virion morphology and major capsid protein structure

Using uranyl acetate, we observed the morphology of *Rhizobium* TM23 virions by negative staining electron microscopy. TM23 likely has an icosahedral appearance of about 25 nm in diameter (Figures 1D–F). The surface features of TM23 seemingly display *mushroom-like* protrusions like those observed in SpV4 (McKenna et al., 1992; Chipman et al., 1998). The protrusions have been associated with an inserted loop within the MCP protein (Roux et al., 2012).

**Figure 3:**
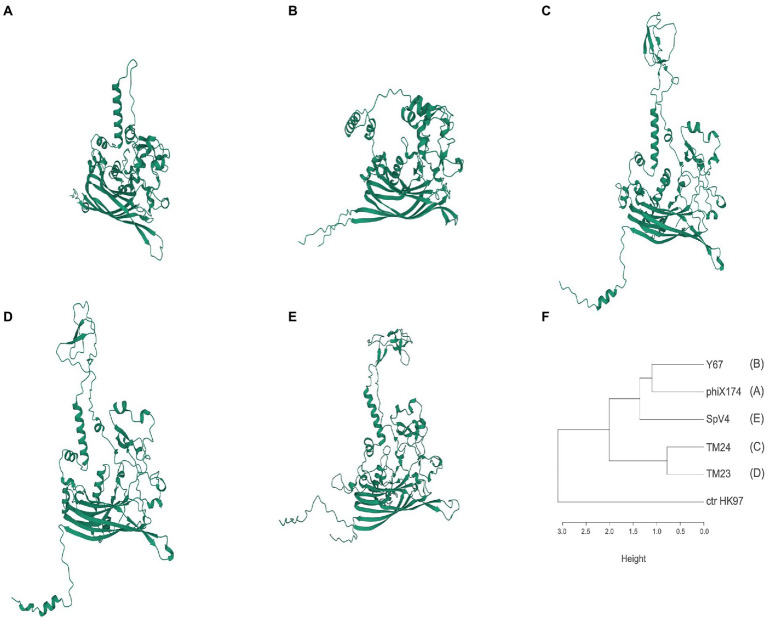
Predicted three-dimensional structure models of MCPs. **(A)** PhiX174; **(B)** Y67 (representing cluster ANIm F40). **(C)** TM24 (F08); **(D)** TM23 (F02); **(E)** SpV4 (*Spiroplasma,* Goshukovirus); **(F)** MCP similarity according to the RMSD values from the superposition between pairs of models (Supplementary Table S4; Supplementary Movies S1–S3). Models were performed with the alphaFold 2.1 program (Jumper et al., 2021).

To search for structural differences in the surface of TM24, TM23, and Y67 phages, we made 3D models of the MCP proteins with AlphaFold2 and compared them with the MCP 3D models of SpV4 and phiX174, and the solved crystal structure of MCP-phiX174 (McKenna et al., 1992). MCP-TM24 and MCP-TM23 have a similar length of 556 and 554 amino acids, respectively, while the MCP-Y67 is minor with 425 amino acids in length. The 3D models made with AlphaFold2 predicted a core of eight antiparallel β-sheets and a variable number of α-helix and loops, consistent with the MCP of SpV4 and phiX174, both 3D model and solved structure (Figure 3). In addition, low IDDT scores were observed between amino acid positions 200–300 (Supplementary Figure S2), a region that corresponds to the large loop followed in the 3D models of MCP-TM23, TM24, and SpV4 but not in the MCP-phiX174 (3D model and structure), where the loop is short, and in MCP-Y67 which lacks a loop in this region, which may be part of the reason why its host range differs from other phages (Supplementary Figure S3).

The superposition of the 3D models assessed by RMSD (Root Median Standard Deviation) showed that MCP-Y67 has a lower RMSD (11.8 A) than MCP-TM23 and TM24 (16.8 and 18.9 A, respectively) when compared with MCP-phiX174 3D model (Supplementary Movie S1–S3). Similarly, the RMSDs between MCP-TM23 and MCP-TM24 3D models are more similar than those with MCP-Y67 (Supplementary Table S4). Although MCP-Y67 is more like the SpV4 model in the core of β-sheets, the large loop made them different. The dendrogram in Figure 3F shows the similarities between the models according to the RMSD. The comparison between the crystallographic structure of the MCP of phiX174 and the AlphaFold2 model obtained from the same protein, indicates RMSD values of 1,267 angstroms. Equivalent RMSD values were observed between the 3D models of the MCPs of microviruses TM23, TM24, and Y67 compared to either the crystallographic structure of MCP-phiX174 or its AlphaFold2 model (Supplementary Table S4).

### Microvirus viral clusters

To look for identities below the ANI threshold of 70% that we used in previous ANIm and ANIb assessments, we made whole genome amino acid comparisons (AAI; Supplementary Figures S4, S5). The three comparative methods detected the largest *Microviridae* cluster, designated as F02. It consisted of 24 *Rhizobium Microviridae* genomes and the *Microviridae*-sp-isolate-ctcf-4 (MH616837.2) that was identified in metagenomes of animal samples, and it was the most divergent genome in the F02 cluster (52% AAI; Lopez et al., 2022). F02 comprises genomes of 6 kb and GC content of about 58–59% related by nucleotide identities of 95% on average and AAI above 60%. The F02 cluster contains four subgroups: (1) The main group (*n* = 20), (2) a group that includes solely Argentinian phages (*n* = 2; AAI ≥ 98%; ANIb = 93%, when compared to other F02 members), (3) the novel phage X92 (AAI = 80%, ANIb = 74%), and (4) RHEph17 (AAI > 98%, ANIb = 93%).

ANIb and AAI comparisons revealed two small clusters consistent with the previous F08 and F40 ANIm clusters. They consist of three microviruses of 6.2 Kb (GC 59%) and two microviruses of 4.7–4.8 kb (GC 57–58%), respectively. In addition, by AAI comparisons, phages (microvirus sp. 1712115-248 and 166) isolated from a sewage oxidation pond and two phages (microvirus Tbat2-88 and 91) isolated from feces samples of the flying fox bat were included in the F08 cluster at AAI 60% (Lopez et al., 2022). The three clusters described here showed little to no ANIb or AAI relationship with genomes of previously described *Microviridae* subfamilies.

**Figure 4:**
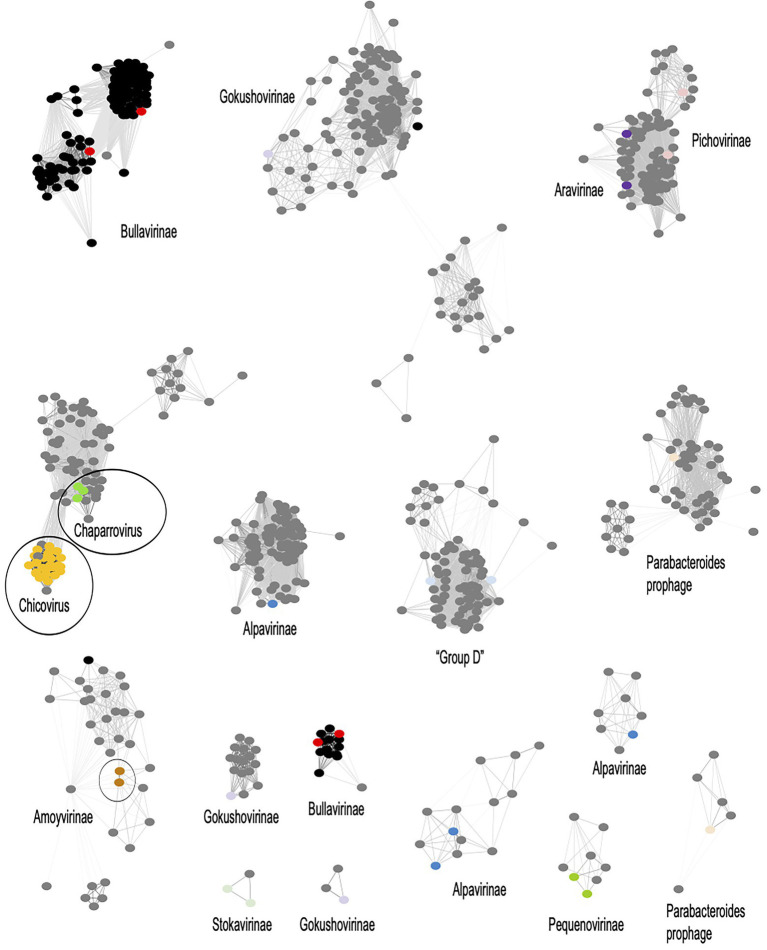
Viral clusters (VCs) of *Microviridae* phages. The network performed with Cytoscape v3.8.2 (https://cytoscape.org/) represents the genomic relationships of *Microviridae* as were inferred from vConTACT-v2. Nodes represent genomes, and the edges connecting nodes are the scores of protein shared clusters, including similarity measures and ICTV inferences. Black dots are microviruses cultivated isolates; grey dots are microviruses identified in metagenomic samples. *Microviridae* subfamilies are named in the corresponding VC, and a color dot within the cluster identifies the subfamily in the MCP phylogenetic tree in Figure 5. Encircled are the *Rhizobium* microviruses within a VC supercluster.

To uncover the genomic relationships of *Rhizobium* microviruses with the other Microviridae subfamilies, we used a network method based on scoring the protein-sharing families between genomes (Jang et al., 2019). The dataset of 2,176 microvirus genomes was included in the vConTACT v2 database (see “Materials and Methods” section). Most of the microvirus genomes (73.5%) in such a database come from metagenomic samples of the gut and feces of humans and other mammals and tortoises (Supplementary Table S3; Orton et al., 2020; Lopez et al., 2022). A small subset of the microvirus genomes included in the database has been determined from viruses isolated *in vivo* by infecting a few bacterial species (Supplementary Table S3). The microviruses are distributed in the vConTACT v2 network (Figure 4; Supplementary Figure S6) in 16 viral clusters (VCs) corresponding to *Microviridae* subfamilies; one is the *Bullavirinae*, which group the phages related to phiX174 and was found in two separated unrelated VCs in the network. Other *Microviridae* subfamilies also appear distributed in two or three VCs. For instance, *Gokushovirinae* and *Alpavirinae* are contained in two and three VCs. Although unexpected, multiple VC clusters in the same subfamily reflect the wide diversity of microviruses within the already defined taxons.

The F02 and F08 *Rhizobium* microviruses were embedded into a VC supercluster of *Microviridae* genomes identified in metagenomic samples (encircled in Figure 4). They are undescribed taxons of *Microviridae* that we propose to name chicoviruses (F02) and chaparroviruses, respectively (F08; see below).

The *Rhizobium* microviruses N39 and Y67 of the F40 cluster were included in the vConTACT-2 network within the *Amoyvirinae* subfamily even though they are far related to vB Cib; an isolate that infects the marine α-proteobacteria *Citromicrobium* (Zheng et al., 2018), to a prophage in the genome of *Novospinghobium* NBRC 16725 from sewage sludge, and with metagenomic samples of marine origin (SOG00694).

### Microviridae phylogenetic lineages

**Figure 5:**
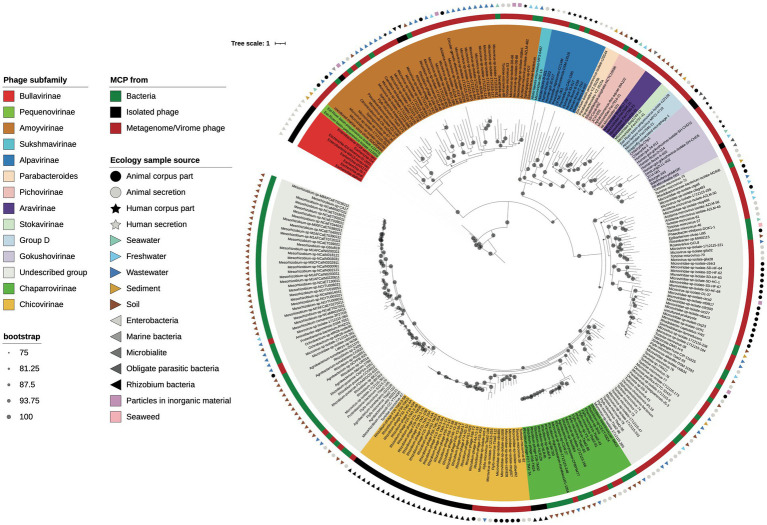
MCP phylogeny. Maximum likelihood phylogenetic tree based on the major capsid protein (MCP) sequences of the *Rhizobium* microviruses, related BlastP hits, and representative phages from different proposed *Microviridae* subfamilies. Bootstrap scores greater than 75% are indicated with a black dot. Phage names are highlighted with colors corresponding to subfamily identity (see color legend to the left).

To investigate the genetic relatedness between *Rhizobium* microviruses and *Microviridae* subfamilies that either have been recognized by the ICTV or have been proposed in previous studies, we constructed a maximum-likelihood (ML) phylogenetic tree based on amino acid sequences of the major capsid protein (MCP; Figure 5). First, MCPs were obtained from microviruses belonging to known subfamilies and metagenomes and isolated microviruses from the GenBank database, and MCPs in bacterial genomes (as part of intact or degenerated prophages) were available on GenBank using BLASTp searches (Supplementary Table S5). MCPs are highly divergent in amino acid sequence, indicating an extensive evolutionary history. The MCP phylogeny yields four major clades. One clade (I) leads to *Bullavirinae*; the second (II), represented by *Amoyvirinae*; a third (III), that contains the *Gokushovirinae* subfamilies and most of the proposed subfamilies from metagenomic samples; and the fourth (IV), large and divergent major clade that includes unknown subfamilies distant from any recognized subfamily of *Microviridae* (Figure 5).

Then, within the clade IV, the *Rhizobium* microviruses that belonged to the VCs of the chicoviruses (F02) and chaparroviruses (F08) were clustered with MCPs of phages detected in fecal samples of vertebrates (Orton et al., 2020; Lopez et al., 2022), and with putative prophages in hosts closely related to *Rhizobium* (*Mesorhizobium*, *Agrobacterium*, *Ochrobactrum*, and *R. pusense*; Figure 5).

Two *Rhizobium* microviruses (ANIm cluster F40) were grouped with the uncultured marine virus clone SOG00694 and various phages and prophages associated with Sphingomonadaceae and other proteobacteria (Figure 5), previously described as *Amoyvirinae* (Zheng et al., 2018).

### Microvirus-host coevolution

Blast searches with the MCP sequences of the three *Rhizobium Microviridae* clusters showed similarities with phages detected in diverse ecological niches, including metagenome samples from animal-associated microbiomes (Orton et al., 2020; Tisza et al., 2021; Lopez et al., 2022), but also from a sewage oxidation pond (Kraberger et al., 2021). MCPs were also found in genomes of bacteria associated with soil and aquatic environments suggesting lysogenic interaction with microviruses (Figure 5). The ample genetic and ecological diversification of microviruses indicates that the phylogenetic history of microviruses may be discordant with the phylogeny of the bacterial host they infect. To investigate this possibility, we compared the topologies of the 16S phylogeny bacterial host with the MCP phylogenies using ParaFit (Legendre et al., 2002).

Moreover, most of these soil bacteria were closely related to *Rhizobium*, within the order of Hyphomicrobiales. In contrast, bacteria with more distantly related MCPs, like those from aquatic environments, were more distantly related to *Rhizobium*.

**Figure 6:**
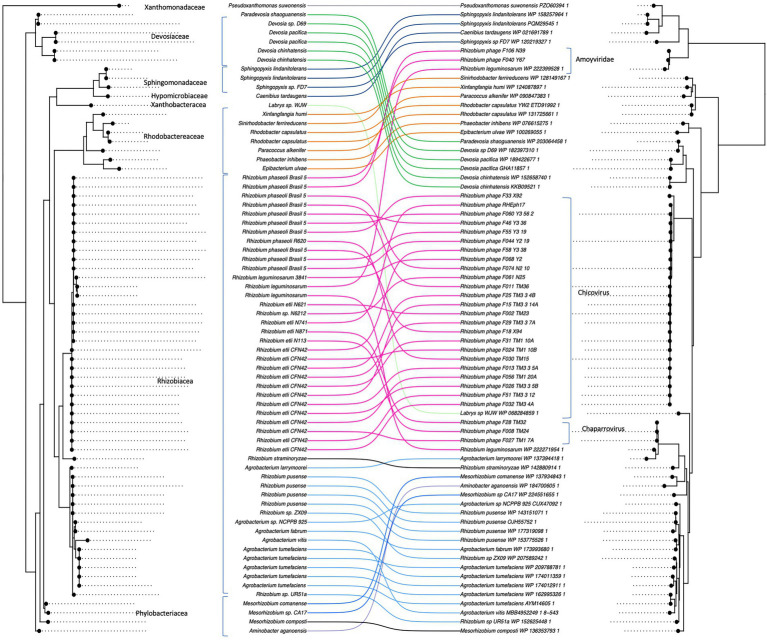
Co-phylogeny between microviruses and their bacterial hosts. Phage major capsid protein (MCP) phylogeny was compared to the bacterial 16S sequence phylogeny with Parafit (Legendre et al., 2002). The lines between the two phylogenies connect phages with their corresponding hosts.

The 16S phylogeny of the MCP-housing bacteria, for which we could obtain species identity and/or were available in the RefSeq database, was significantly similar to the MCP phylogeny (i.e., co-phylogeny ParaFit: *p* = 3e-04) since genetically similar MCPs were shared among genetically similar bacterial hosts (Figure 6). Although the presence of MCP alone does not necessarily indicate the presence of microvirus prophages, the result of single-marker phylogenies suggested that microviruses have coevolved with their bacterial host.

## Discussion

Knowledge of the abundance and diversity of the *Microviridae* family is expanding, mainly due to the resolution of metagenomic methods and powerful bioinformatics algorithms. Metagenomics has allowed the identification and classification of single-stranded DNA viruses in DNA obtained from various ecosystems (Kirchberger et al., 2022). In contrast, the isolation of viral particles of microviruses by bacterial infection has progressed slowly. Cultivated microviruses constitute 16% of the complete *Microviridae* genomes represented in GenBank. Furthermore, most known isolated microviruses are relatives of phiX174 (*Bullavirinae*), infecting Enterobacteria (Brentlinger et al., 2002). In this work, a comprehensive collection of cultivated microvirus isolates that infect the symbiotic bacterium *Rhizobium* was placed in a phylogenetic context revealing two novel lineages and one included within the already proposed subfamily *Amoyvirinae* (Zheng et al., 2018). The new lineages are unrelated to recognized ICTV *Microviridae* subfamilies (*Bullavirinae* and *Gokushovirinae*) and other proposed subfamilies (Krupovic and Forterre, 2011; Roux et al., 2012; Quaiser et al., 2015; Zheng et al., 2018). The biological properties of representative *Rhizobium* microviruses and the proposed taxonomic affiliation reported here make them a valuable resource for further experimental studies.

The diversity of bacteriophages infecting soil rhizobia, well-studied and economically significant bacteria, such as *Rhizobium*, remains underexplored. Until recently, *Caudovirales* were the only group of phages known to infect rhizobia (Werquin et al., 1988; Santamaría et al., 2014, 2022). Recently, a collection of 63 lytic ssDNA viruses was isolated from agricultural soils and showed abundances ranging from 0 to 60%, predominating over *Caudovirales* in one field (Van Cauwenberghe et al., 2021). The genome sequence of 29 microviruses, representative of the entire collection, shows the basic genetic structure of most microviruses with genes encoding for the capsid (*mcp*), the replication (*rep*), and the endolysin (*lys*). The genes encoding for the pilot protein and other auxiliary proteins for capsid assembly and replication, as in *Bullavirinae* and *Gokushovirinae* members, were undetected in our BlastP searches. Though the pilot protein has been involved in the superinfection exclusion of phages of *Gokushovirinae*, it has remote sequence similarity with sequences of microviruses of other subfamilies (Krupovic and Forterre, 2011; Kirchberger et al., 2021; Zucker et al., 2022).

We describe *Rhizobium* microviruses clustered in three distinct groups based on ANIb, AAI, protein-sharing networks, and MCP phylogeny. Two novel *Microviridae* clusters were included within an ample viral cluster of uncultivated microviruses of metagenomic origin (Figure 4). We refer to these as chaparroviruses and chicoviruses from the synonymous words chaparro- and chico-, meaning “small in size” in Spanish. They formed groups distinct from any previously described subfamilies of *Microviridae*. Chaparroviruses and chicoviruses are sister clades that share similar proteins yet form separate clusters in protein-sharing networks and show low nucleotide identity (ANIb <70%). Chaparroviruses share about 98% ANIm, and chicoviruses share 95% ANIm. This difference in cohesion is due to the presence of four subclusters within the chicoviruses, which partially reflect the different spatial origins of the phages. The phylogeny of the MCP protein confirms the relationships between chaparroviruses and chicoviruses and various undescribed clades, highlighting how little is known about *Microviridae* diversity.

The third group of *Rhizobium* microviruses, represented by two phages, is included within the *Amoyvirinae* subfamily, a recently described group of lytic phages and prophages infecting marine Sphingomonadaceae (Zheng et al., 2018). *Amoyvirinae* have notably smaller genomes than most *Microviridae*, about 4.3 kb vs. about 6 kb, yet those infecting rhizobia appear to have larger genomes of approximately 4.8 kb. The two *Rhizobium Amoyvirinae* share nearly 84% ANIm and form a coherent viral cluster within the protein-sharing network. In addition, these phages are far related to vB_Cib of *Citromicrobium*, another cultivated representant of *Amoyvirinae* of marine origin (Zheng et al., 2018).

The MCP structure differs among microvirus subfamilies (Roux et al., 2012; Quaiser et al., 2015). Three-dimensional models of the MCP of *Rhizobium* microviruses showed a *jelly-roll* fold, commonly found in capsids of microviruses and RNA and ssDNA virus of eukaryotes (Krupovic and Koonin, 2017). The 3D models of the MCP of the phages TM23 (chicovirus) and TM24 (chaparrovirus) are similar to each other and to the published MCP 3D models of Gokushovirus, Alphavirus, Pichovirus, Stokavirus, and Aravirus (Roux et al., 2012; Quaiser et al., 2015). They have a large variable loop at the same position in the structure of phiX174, which in contrast, has a small loop (McKenna et al., 1992). Although the model of MCP-Y67 (*Amoyvirinae*) is reminiscent of MCP-phiX174, its loop is reduced to a hypervariable region. The alignment of the amino acid sequence of the MCP-TM23 and MCP-Y67 showed a region from the proline 205 to glycine 310 in the first protein that is absent in the last (Supplementary Figure S3). Comparing the MCP models suggests that the hypervariable loop may have a role in the host recognition and diversification of the *Microviridae*.

A key element to understanding the diversification and adaptation of microviruses is the identification of their bacterial hosts and their range of infection. Nevertheless, most microviruses genomes come from metagenomes from various ecological niches, and there is limited knowledge about the bacterial species they infect. *Rhizobium* microviruses have a broad host range of infection of diverse strains of *R. etli* and *R. phaseoli*. *Chicoviruses*, the group more represented in the agriculture plot sampled, possibly represents an ongoing evolving lineage specializing in infecting *Rhizobium*. Few members of the currently known subfamilies have been identified in the *Rhizobium Microviridae* (e.g., *Bullavirinae* or *Gokushovirinae*). Few or none of the *Gokushovirinae* are presently known to infect hosts of the superphylum Proteobacteria, suggesting that different clades of *Microviridae* have specialized to infect particular taxa of bacteria or at least are more prevalent in these taxa. Chaparroviruses and chicoviruses infecting *Rhizobium* are closely related to various phages detected mainly in fecal viromes of vertebrates (Tisza et al., 2021; Lopez et al., 2022) whose gut microbiomes are noticeably richer in Proteobacteria (Sun et al., 2020; Kim et al., 2021). Proteobacteria are rarer in human gut microbiomes (Reiss et al., 2016; Senghor et al., 2018) and increased abundances indicate dysbiosis and disease (Morgan et al., 2012; Shin Na-Ri et al., 2015), while Bacteroidota, common hosts of other *Microviridae* (e.g., *Alpavirinae* and *Pichovirinae*), are more dominant (Reiss et al., 2016). Furthermore, MCPs closely related to chaparroviruses and chicoviruses are found as prophages in a small subset of genomes of host strains closely related to *Rhizobium* within the order of Hyphomicrobiales. In contrast, more distantly related MCPs are found in members of the corresponding order of Rhodobacterales (Figures 5,6).

The co-phylogeny of MCPs and 16S sequences suggests coevolutionary history between the *Microviridae* described here and their hosts. The observed coevolutionary pattern may be due to reciprocal adaptation and counter-adaption between the virus and its host or long-term prophage integration. Lysogeny of microviruses has been experimentally demonstrated for synthetic Gokushoviruses, which can integrate into the host genome and produce lytic plaques (Kirchberger and Ochman, 2020). Lysogenic microviruses have also been described in *Alpavirinae* (Krupovic and Forterre, 2011) and *Bullavirinae* (Kirchberger et al., 2021). The discovery of microvirus sequences (e.g., *mcp*) integrated into genomes of some species of bacteroidetes, proteobacteria, and enterobacteria support a lysogenic cycle. Nevertheless, the divergence of microvirus sequences is so great that it has been challenging to demonstrate the presence of complete microvirus prophages. Recent work using HMM profiles and recursive Blast has allowed the identification of *Microviridae* prophages in a vast collection of genomes, including some in Rhizobiaceae (Kirchberger et al., 2022).

Some MCPs related to *Rhizobium Microviridae* could be leftovers from degenerated prophages, also called cryptic prophages (Casjens, 2003). In the long term, footprints of the interaction between microviruses and their hosts remain in the genomes. Microviruses may have been initially integrated before the divergence of their respective hosts and got stranded due to a deleterious mutation (Wang et al., 2010; Bobay et al., 2014). However, prophages and cryptic prophages are expected to degenerate rapidly out of existence since they represent a metabolic cost (Campbell, 1998) unless they experience purifying selection due to a benefit they provide to the host (Bobay et al., 2014).

Capsid proteins are generally not retained and are increasingly rare in more degenerated prophages (Khan et al., 2020; Pattenden et al., 2022). Our observation of virion sequences clustering intertwined with prophages indicates relatively recent prophage integrations and continuous dynamic alternations between virulent and temperate lifestyles.

Recent efforts have been made to transform viral taxonomy from a morphology-based into a genome-based classification (Aiewsakun and Simmonds, 2018; Turner et al., 2021). One key aspect of this effort is setting out generalizable and clear delineations of various taxonomic levels (e.g., for *Caudovirales* in Turner et al., 2021). In analogy with such guidelines, we identify two new species within the *Amoyvirinae* and four new species in the chicovirus group. In a recent preprint, the same chaparroviruses, chicoviruses, and related prophages described here were classified as one subfamily named *Occultatumvirinae* (Zucker et al., 2022). Then, this would suggest that chaparroviruses and chicoviruses are two genera within this subfamily. Though both groups are connected in a protein-sharing network, they form distinct clusters and share little average nucleotide identity.

Moreover, various previously suggested subfamilies are either internally divided into multiple monophyletic clusters in the protein-sharing network or are more connected than chaparroviruses and chicoviruses. Turner and coauthors (Turner et al., 2021) proposed to define *Caudovirales* phage families as “cohesive and monophyletic groups in the … proteome-based clustering tools.” This definition does not apply to the current classification of *Microviridae*, indicating the need for a more precise delineation of taxonomic levels within this group. The taxonomy of *Microviridae* could be revised to match delineations set out for *Caudovirales*. One step in this direction would be to elevate *Microviridae* to the rank of order and its subfamilies to families (Kirchberger et al., 2022). However, the difference in genome sizes and evolutionary relevant genomic sizes might challenge the idea of a generalizable classification system.

## Data availability statement

The datasets presented in this study can be found in online repositories. The names of the repository/repositories and accession number(s) can be found in the article/Supplementary material.

## Author contributions

VG: conceptualization, formal analysis, funding acquisition, investigation, methodology, project administration, supervision, validation, visualization, writing—original draft, writing—review and editing. RS: data curation, formal analysis, methodology, software, validation, visualization. PB: data curation, methodology, resources, software. JVC: conceptualization, data curation, formal analysis, investigation, methodology, resources, software, writing—original draft, writing—review and editing. All authors contributed to the article and approved the submitted version.

## Funding

The work was supported by PAPIIT-UNAM 2017–2019 (IN209817 to VG). JVC received Postdoctoral Scholarship from DGAPA-UNAM (2016–2018); JVC was partially supported by National Science Foundation (NSF; DEB-1457508, IOS-1759048, to E.L. Simms).

## Conflict of interest

The authors declare that the research was conducted in the absence of any commercial or financial relationships that could be construed as a potential conflict of interest.

## Publisher’s note

All claims expressed in this article are solely those of the authors and do not necessarily represent those of their affiliated organizations, or those of the publisher, the editors and the reviewers. Any product that may be evaluated in this article, or claim that may be made by its manufacturer, is not guaranteed or endorsed by the publisher.

## Footnotes

^1^https://github.com/FelixKrueger/TrimGalore (Accessed August 24, 2022).

^2^https://cytoscape.org/ (Accessed August 24, 2022).
