# Supplementary material for: Novel lineages of single-stranded DNA phages that coevolved with the symbiotic bacteria Rhizobium
Source: Front Microbiol. 2022 Sep 13;13:990394. doi: 10.3389/fmicb.2022.990394 (PMC9512667; doi:10.3389/fmicb.2022.990394)

Fig. S1

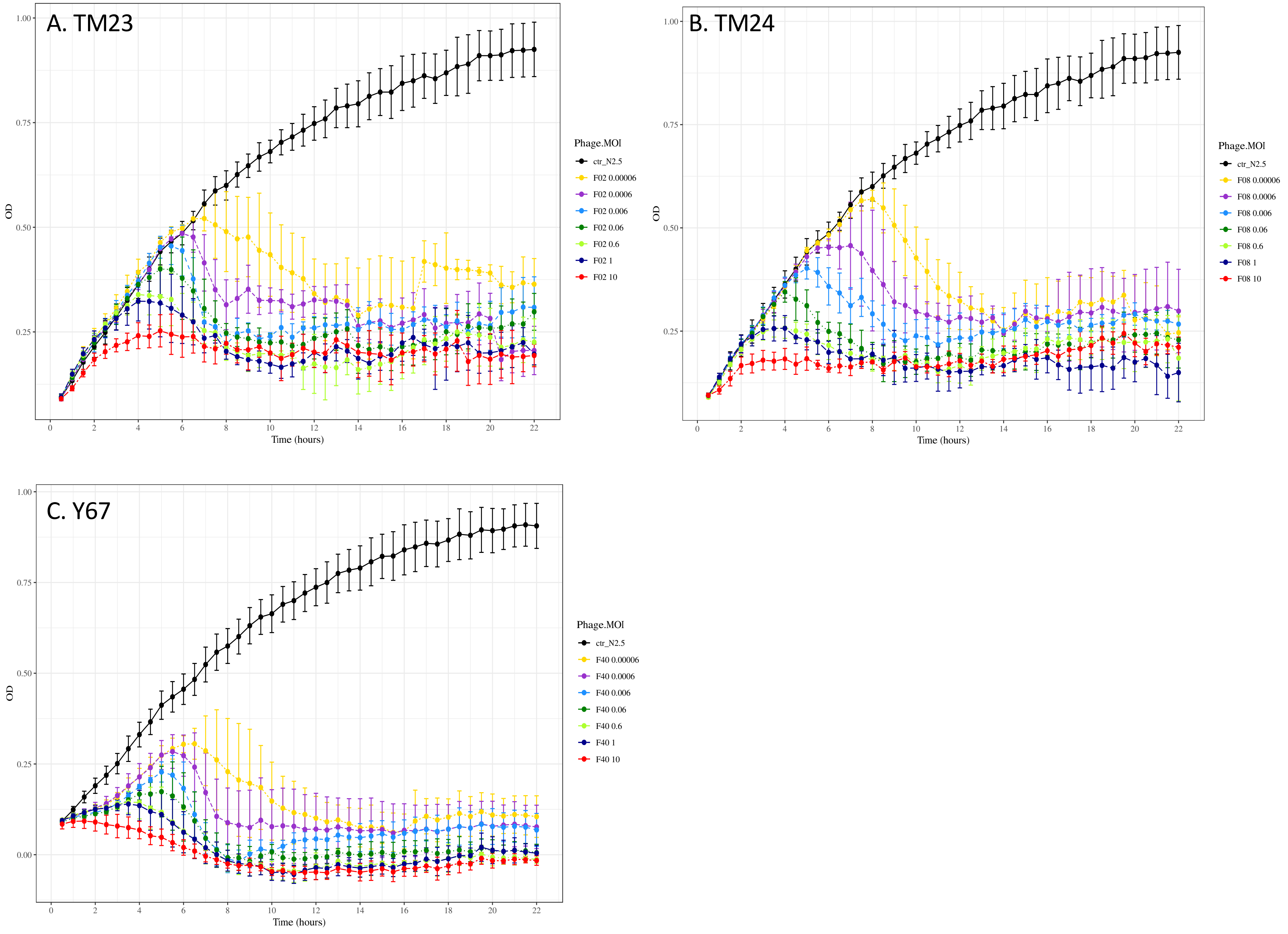
A. TM23
B. TM24
C. Y67
OD
Time (hours)
Phage.MOI
ctr_N2.5
F02 0.00006
F02 0.0006
F02 0.006
F02 0.06
F02 0.6
F02 1
F02 10
F08 0.00006
F08 0.0006
F08 0.006
F08 0.06
F08 0.6
F08 1
F08 10
F40 0.00006
F40 0.0006
F40 0.006
F40 0.06
F40 0.6
F40 1
F40 10
0.00
0.25
0.50
0.75
1.00
0
2
4
6
8
10
12
14
16
18
20
22

Fig. S2

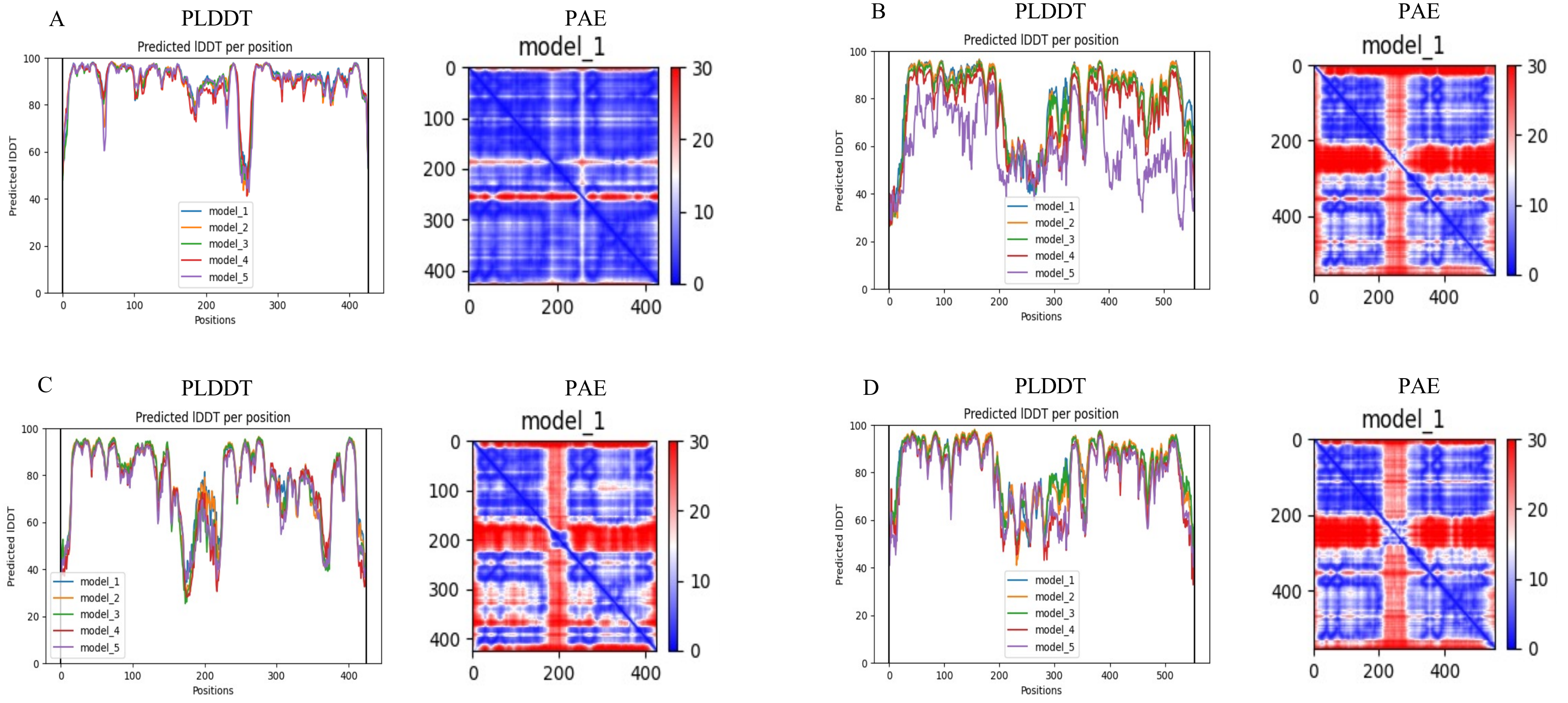
A
PLDDT
Predicted lDDT per position
Predicted lDDT
Positions
model_1
model_2
model_3
model_4
model_5
PAE
model_1
B
PLDDT
Predicted lDDT per position
Predicted lDDT
Positions
model_1
model_2
model_3
model_4
model_5
PAE
model_1
C
PLDDT
Predicted lDDT per position
Predicted lDDT
Positions
model_1
model_2
model_3
model_4
model_5
PAE
model_1
D
PLDDT
Predicted lDDT per position
Predicted lDDT
Positions
model_1
model_2
model_3
model_4
model_5
PAE
model_1

Supp. Fig. S3. ClustalW alignment of the complete amino acid sequence of MCP-TM23 and MCP-Y67. The alignment was obtained from UCSF ChimeraX. Conserved b-sheets regions are in yellow. The loop in MCP-TM23 is coloured in magenta from proline-205 to glycine-310.

```
CLUSTAL W ALN saved from UCSF ChimeraX

TM23_MCP_unrelaxed_model_1      MGCVWCAPSSTGEENMTVNDAAIQTRKMTTPVPVERSRRNHKIRNLTSL. 49
 Y67_MCP_unrelaxed_model_1      .....................MKGVTLARPQKVNRSKIRVPTTNYRLVQ 28
                                                      ::  .:: *  *:**: .    *   :

TM23_MCP_unrelaxed_model_1      PAGKCVPIAVVPLLREDAASMSVSLAFEMQETVEILLNSVNVNVQCWLFP 99
 Y67_MCP_unrelaxed_model_1      RPWQIQPFCIFPVLPGEILRKTLWTDTAITDPLLNRFQGWHCETKFFYVR 78
                                 . :  *:.:.*:*  :    ::     : :.:   ::. : :.: : .

TM23_MCP_unrelaxed_model_1      NLASTRFRSMDDINLSYAGEPRGTDPVVPW...FELAAAGAYGTNAIHDR 146
 Y67_MCP_unrelaxed_model_1      ARD.TVPASSDTIKNIF......IDPDANLSSLYE.......AADAKY.. 112
                                    *   * * *:  :       ** .     :*       .::* :

TM23_MCP_unrelaxed_model_1      LGKHAKSTDMVNMAYVEAYNAIWNHRATEISPNITQRARLDKTLAQAFWP 196
 Y67_MCP_unrelaxed_model_1      ..FHAYGVN.......WLKNCVRQIVENYY......RAE........... 136
                                   ** ..:          *.: :   .        **.

TM23_MCP_unrelaxed_model_1      KNMFSHIVPDFDQAVMEGEVALNVTNQMLPLVKTGASSGINVKFTGGAER 246
 Y67_MCP_unrelaxed_model_1      ...........DEAPDD......YTIDGLWAATVGTD............. 156
                                           *:*  :       * : *  ...*:.

TM23_MCP_unrelaxed_model_1      TLVINAGDNIVRGAGSNFAGTQSITEFTNVFAELQDNGITVSLANIELAR 296
 Y67_MCP_unrelaxed_model_1      ....NWTDSMV.......LGAQQDAEDVSLTVGVDD...AIGMRELNSAQ 192
                                    *  *.:*        *:*. :* ..: . ::*   ::.: ::: *:

TM23_MCP_unrelaxed_model_1      KTQAWADIRKQYNGLSEEMLIDLLMD.GISVPEQMWK.QPILLKAAQTQF 344
 Y67_MCP_unrelaxed_model_1      RMYEFLKM....GLLTDMDYSDFLRTYGVKIPAEELPGKPKYLGGVSEWT 238
                                :   : .:    . *::    *:*   *:.:* :    :*  * ...

TM23_MCP_unrelaxed_model_1      GMAKRYASNGEALTQSVVNGAAAVEFRVATPK...VPCGGVIMITAEIAP 391
 Y67_MCP_unrelaxed_model_1      MPTNTVEP.....TTGVATTACYWSNKDRLDKDHLFKEPGFVVGLRVYRP 283
                                  ::   .     * .*.. *.  . :    *   .   *.::      *

TM23_MCP_unrelaxed_model_1      EQLFERRLDPY...LHAQGVADLPSYTRDFLDPE.KVDVVPNRYIDIDHD 437
 Y67_MCP_unrelaxed_model_1      .KIFRSGIKGSVSGIMDTALEWLPAVLMD..DPDTSLTRVASGAGPLG.A 329
                                 ::*.  :.     :   .:  **:   *  **: .:  *..    :.

TM23_MCP_unrelaxed_model_1      TPTDVYGYAPLN.YLWNDAGPGIGGRFIR.....PTVDASFDEDRQAIWA 481
 Y67_MCP_unrelaxed_model_1      LGQD.YTFDMRDYFLYGEEFMNFDPTTWSGAVALPAVETAPTPD.....K 373
                                   * * :   : :*:.:   .:.          *:*:::   *

TM23_MCP_unrelaxed_model_1      VETQNPTLS.EDAYLVPADIHLKPFWTSTIDPFDCITIGETVITG...NT 527
 Y67_MCP_unrelaxed_model_1      FNKRYPSLAMAKQLFVDATADVK.......TRIETSGRVDMTIAGRVIDT 416
                                .:.: *:*:  .  :* *  .:*         ::     : .*:*   :*

TM23_MCP_unrelaxed_model_1      VFGPRLIEAQAASDYDKVMEKVDVARIEK 556
 Y67_MCP_unrelaxed_model_1      TPSVARLEV.................... 425
                                . .   :*.
```

Fig. S4

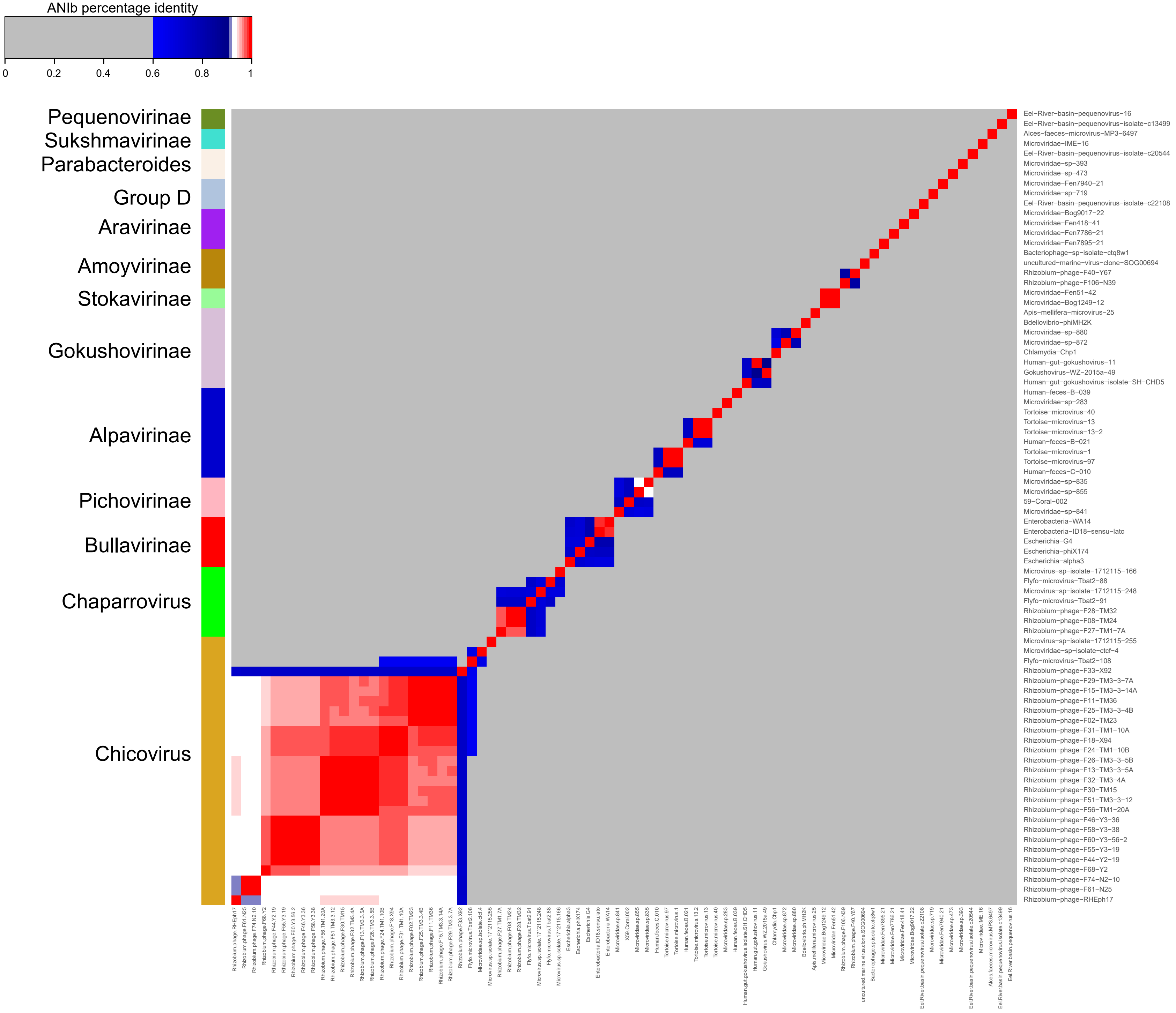
ANIb percentage identity
0
0.2
0.4
0.6
0.8
1
Pequenovirinae
Sukshmavirinae
Parabacteroides
Group D
Aravirinae
Amoyvirinae
Stokavirinae
Gokushovirinae
Alpavirinae
Pichovirinae
Bullavirinae
Chaparrovirus
Chicovirus
Eel−River−basin−pequenovirus−16
Eel−River−basin−pequenovirus−isolate−c13499
Alces−faeces−microvirus−MP3−6497
Microviridae−IME−16
Eel−River−basin−pequenovirus−isolate−c20544
Microviridae−sp−393
Microviridae−sp−473
Microviridae−Fen7940−21
Microviridae−sp−719
Eel−River−basin−pequenovirus−isolate−c22108
Microviridae−Bog9017−22
Microviridae−Fen418−41
Microviridae−Fen7786−21
Microviridae−Fen7895−21
Bacteriophage−sp−isolate−ctq8w1
uncultured−marine−virus−clone−SOG00694
Rhizobium−phage−F40−Y67
Rhizobium−phage−F106−N39
Microviridae−Fen51−42
Microviridae−Bog1249−12
Apis−mellifera−microvirus−25
Bdellovibrio−phiMH2K
Microviridae−sp−880
Microviridae−sp−872
Chlamydia−Chp1
Human−gut−gokushovirus−11
Gokushovirus−WZ−2015a−49
Human−gut−gokushovirus−isolate−SH−CHD5
Human−feces−B−039
Microviridae−sp−283
Tortoise−microvirus−40
Tortoise−microvirus−13
Tortoise−microvirus−13−2
Human−feces−B−021
Tortoise−microvirus−1
Tortoise−microvirus−97
Human−feces−C−010
Microviridae−sp−835
Microviridae−sp−855
59−Coral−002
Microviridae−sp−841
Enterobacteria−WA14
Enterobacteria−ID18−sensu−lato
Escherichia−G4
Escherichia−phiX174
Escherichia−alpha3
Microvirus−sp−isolate−1712115−166
Flyfo−microvirus−Tbat2−88
Microvirus−sp−isolate−1712115−248
Flyfo−microvirus−Tbat2−91
Rhizobium−phage−F28−TM32
Rhizobium−phage−F08−TM24
Rhizobium−phage−F27−TM1−7A
Microvirus−sp−isolate−1712115−255
Microviridae−sp−isolate−ctcf−4
Flyfo−microvirus−Tbat2−108
Rhizobium−phage−F33−X92
Rhizobium−phage−F29−TM3−3−7A
Rhizobium−phage−F15−TM3−3−14A
Rhizobium−phage−F11−TM36
Rhizobium−phage−F25−TM3−3−4B
Rhizobium−phage−F02−TM23
Rhizobium−phage−F31−TM1−10A
Rhizobium−phage−F18−X94
Rhizobium−phage−F24−TM1−10B
Rhizobium−phage−F26−TM3−3−5B
Rhizobium−phage−F13−TM3−3−5A
Rhizobium−phage−F32−TM3−4A
Rhizobium−phage−F30−TM15
Rhizobium−phage−F51−TM3−3−12
Rhizobium−phage−F56−TM1−20A
Rhizobium−phage−F46−Y3−36
Rhizobium−phage−F58−Y3−38
Rhizobium−phage−F60−Y3−56−2
Rhizobium−phage−F55−Y3−19
Rhizobium−phage−F44−Y2−19
Rhizobium−phage−F68−Y2
Rhizobium−phage−F74−N2−10
Rhizobium−phage−F61−N25
Rhizobium−phage−RHEph17

Fig. S5

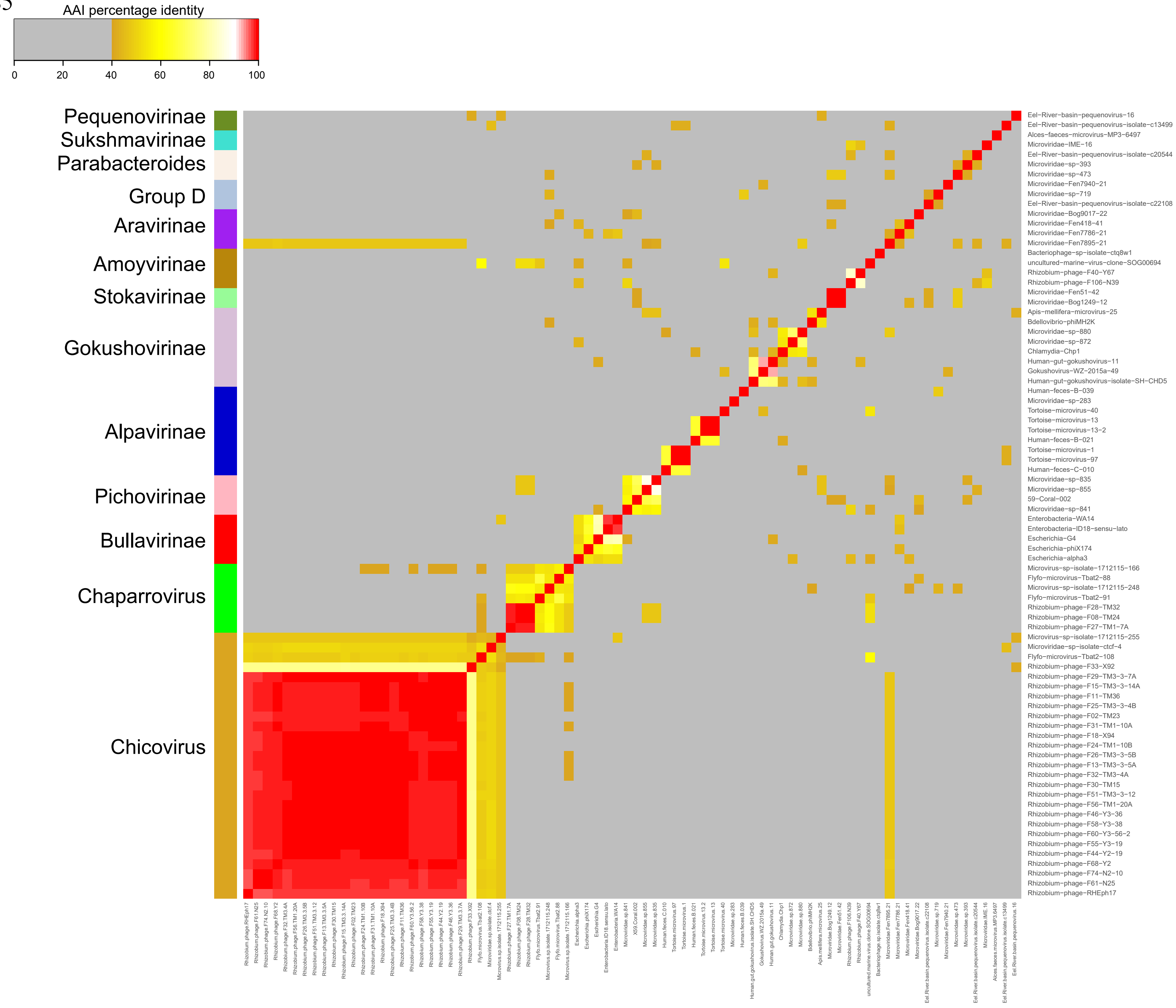

AAI percentage identity
0
20
40
60
80
100
Pequenovirinae
Sukshmavirinae
Parabacteroides
Group D
Aravirinae
Amoyvirinae
Stokavirinae
Gokushovirinae
Alpavirinae
Pichovirinae
Bullavirinae
Chaparrovirus
Chicovirus
Eel−River−basin−pequenovirus−16
Eel−River−basin−pequenovirus−isolate−c13499
Alces−faeces−microvirus−MP3−6497
Microviridae−IME−16
Eel−River−basin−pequenovirus−isolate−c20544
Microviridae−sp−393
Microviridae−sp−473
Microviridae−Fen7940−21
Microviridae−sp−719
Eel−River−basin−pequenovirus−isolate−c22108
Microviridae−Bog9017−22
Microviridae−Fen418−41
Microviridae−Fen7786−21
Microviridae−Fen7895−21
Bacteriophage−sp−isolate−ctq8w1
uncultured−marine−virus−clone−SOG00694
Rhizobium−phage−F40−Y67
Rhizobium−phage−F106−N39
Microviridae−Fen51−42
Microviridae−Bog1249−12
Apis−mellifera−microvirus−25
Bdellovibrio−phiMH2K
Microviridae−sp−880
Microviridae−sp−872
Chlamydia−Chp1
Human−gut−gokushovirus−11
Gokushovirus−WZ−2015a−49
Human−gut−gokushovirus−isolate−SH−CHD5
Human−feces−B−039
Microviridae−sp−283
Tortoise−microvirus−40
Tortoise−microvirus−13
Tortoise−microvirus−13−2
Human−feces−B−021
Tortoise−microvirus−1
Tortoise−microvirus−97
Human−feces−C−010
Microviridae−sp−835
Microviridae−sp−855
59−Coral−002
Microviridae−sp−841
Enterobacteria−WA14
Enterobacteria−ID18−sensu−lato
Escherichia−G4
Escherichia−phiX174
Escherichia−alpha3
Microvirus−sp−isolate−1712115−166
Flyfo−microvirus−Tbat2−88
Microvirus−sp−isolate−1712115−248
Flyfo−microvirus−Tbat2−91
Rhizobium−phage−F28−TM32
Rhizobium−phage−F08−TM24
Rhizobium−phage−F27−TM1−7A
Microvirus−sp−isolate−1712115−255
Microviridae−sp−isolate−ctcf−4
Flyfo−microvirus−Tbat2−108
Rhizobium−phage−F33−X92
Rhizobium−phage−F29−TM3−3−7A
Rhizobium−phage−F15−TM3−3−14A
Rhizobium−phage−F11−TM36
Rhizobium−phage−F25−TM3−3−4B
Rhizobium−phage−F02−TM23
Rhizobium−phage−F31−TM1−10A
Rhizobium−phage−F18−X94
Rhizobium−phage−F24−TM1−10B
Rhizobium−phage−F26−TM3−3−5B
Rhizobium−phage−F13−TM3−3−5A
Rhizobium−phage−F32−TM3−4A
Rhizobium−phage−F30−TM15
Rhizobium−phage−F51−TM3−3−12
Rhizobium−phage−F56−TM1−20A
Rhizobium−phage−F46−Y3−36
Rhizobium−phage−F58−Y3−38
Rhizobium−phage−F60−Y3−56−2
Rhizobium−phage−F55−Y3−19
Rhizobium−phage−F44−Y2−19
Rhizobium−phage−F68−Y2
Rhizobium−phage−F74−N2−10
Rhizobium−phage−F61−N25
Rhizobium−phage−RHEph17

Fig. S6

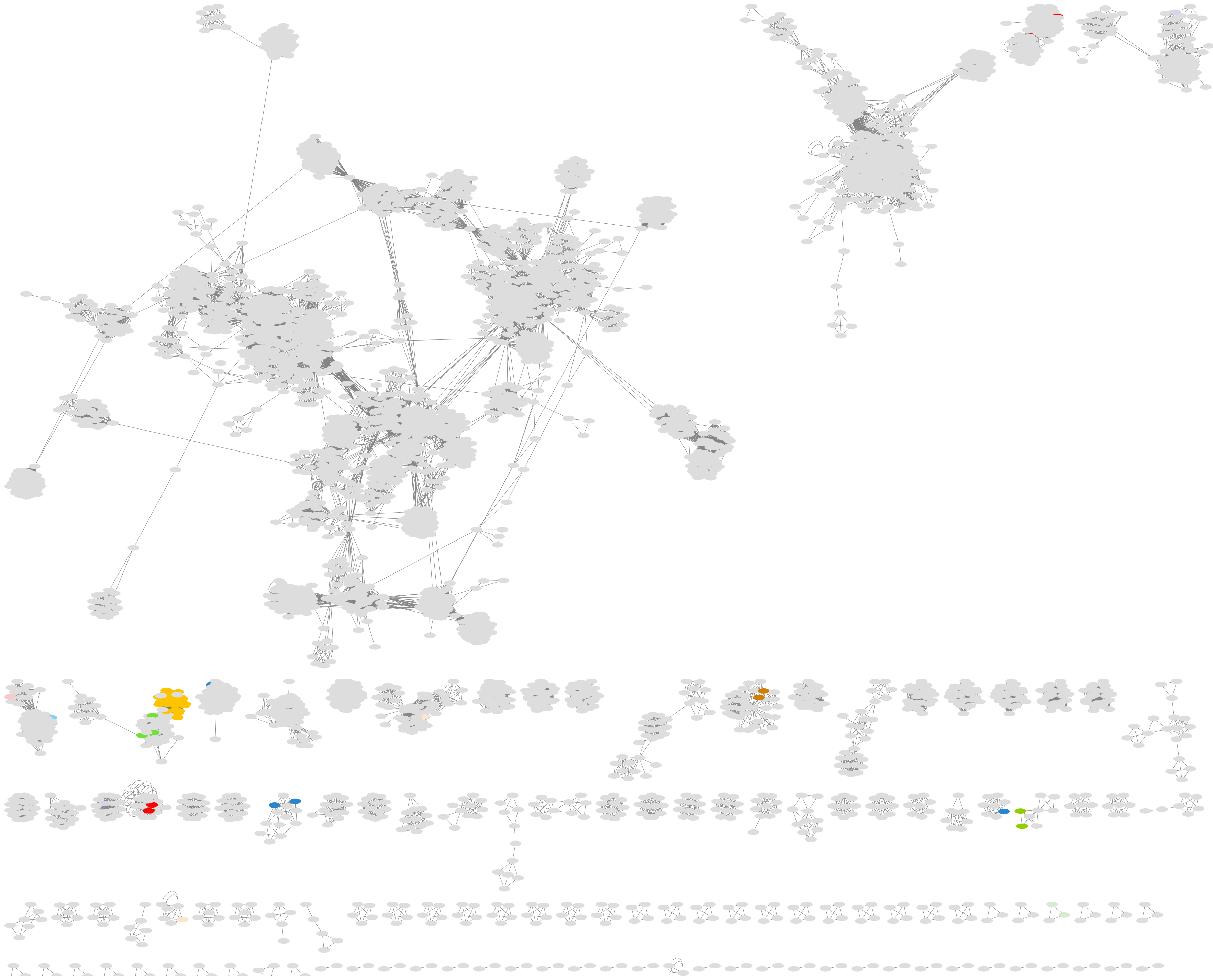

Supplement: Supplementary file 9 [file Presentation_1.pdf]
